# Polyimide Electrode-Based Electrical Stimulation Impedes Early Stage Muscle Graft Regeneration

**DOI:** 10.3389/fneur.2019.00252

**Published:** 2019-03-22

**Authors:** Shriya Srinivasan, Keval Vyas, Malia McAvoy, Peter Calvaresi, Omar F. Khan, Robert Langer, Daniel G. Anderson, Hugh Herr

**Affiliations:** ^1^Harvard/MIT Health Sciences and Technology, Harvard Medical School, Boston, MA, United States; ^2^Center for Extreme Bionics, Massachusetts Institute of Technology, Cambridge, MA, United States; ^3^David H. Koch Institute for Integrative Cancer Research, Massachusetts Institute of Technology, Cambridge, MA, United States; ^4^Department of Chemical Engineering, Massachusetts Institute of Technology, Cambridge, MA, United States; ^5^Institute for Medical Engineering and Science, Massachusetts Institute of Technology, Cambridge, MA, United States

**Keywords:** electrical stimulation, muscle regeneration, nerve regeneration, free flap, reinnervation, neurophysiology

## Abstract

Given the increasing use of regenerative free muscle flaps for various reconstructive procedures and neuroprosthetic applications, there is great interest and value in their enhanced regeneration, revascularization, and reinnervation for improved functional recovery. Here, we implant polyimide-based mircroelectrodes on free flap grafts and perform electrical stimulation for 6 weeks in a murine model. Using electrophysiological and histological assessments, we compare outcomes of stimulated grafts with unstimulated control grafts. We find delayed reinnervation and abnormal electromyographic (EMG) signals, with significantly more polyphasia, lower compound muscle action potentials and higher fatigability in stimulated animals. These metrics are suggestive of myopathy in the free flap grafts stimulated with the electrode. Additionally, active inflammatory processes and partial necrosis are observed in grafts stimulated with the implanted electrode. The results suggest that under this treatment protocol, implanted epimysial electrodes and electrical stimulation to deinnervated, and devascularized flaps during the early recovery phase may be detrimental to regeneration. Future work should determine the optimal implantation and stimulation window for accelerating free muscle graft regeneration.

## Introduction

Peripheral nerve regeneration is a promising and important area of clinical development and plays an integral role in the functionality of reinnervated organs (1–3). Repair for many brachial plexus, spinal cord, and reconstructive limb surgeries hinges heavily on peripheral nerve and muscle free flap regeneration (4). In the case of organ transplants, speedy reinnervation of target organs remains a concern that limits certain transplant applications (5). With the increase of peripheral nerve reconstruction and advent of recent techniques employing regenerative muscle and nerve constructs for traumatic and oncological reconstruction (6–8), and myoelectric device control (9–11), there is a great need to optimize regeneration (11–13).

Regenerative muscle grafts have garnered increasing importance and interest in the field of neuroprostheses for limb control (9, 11, 14). A devascularized muscle graft can be neurotized using a transected peripheral nerve to create a regenerative peripheral nerve interface (RPNI). Given that these muscle grafts reinnervate, revascularize, and produce stable electromyographic (EMG) signals, electrodes can be placed on each muscle graft, and naturally generated EMG signals can be used to drive myoelectric limb prostheses (11, 12). However, the time course of reinnervation (>2 months for murine and >3 months for primate models) and regeneration (~50% atrophy at 6 months) are suboptimal (9, 11, 12). Neural plasticity studies have shown that cortical atrophy and reorganization can take place as early as 2 weeks (15). Thus, to preserve appropriate function, it is necessary to have early reinnervation and regeneration to mitigate unwanted reorganization.

Functional electrical stimulation (FES) has been widely reported to improve regeneration of nerve and muscle tissue (3, 16–18). Both acute and chronic stimulation approaches have shown improved axonal regeneration, and healing time courses (16, 19, 20). FES of nerves upregulates the expression of neurotrophic factors [i.e., brain-derived neurotrophic factor (BDNF), and glial cell–derived neurotrophic factor (GDNF)], and also enhances nerve growth factor (NGF)-induced signaling and gene expression, specifically the MEK–ERK1/2 pathway and Egr1 expression, accelerating neoaxogenesis, neurite outgrowth, and sprouting (21–23). For myocyte regeneration, FES upregulates the production of vascular endothelial growth factor (VEGF) and insulin-like growth factor (IGF), which are important in muscle regeneration (24, 25). Moreover, by increasing myogenic precursor cell proliferation and fusion with mature myofibers, FES improves the regenerative capacity of skeletal muscle (26, 27). Electrical stimulation has widely shown benefit for revascularization through the induction of angiogenic factors such as VEGF and angiotensin II (28, 29). Regenerative muscle grafts are relatively fragile given their deinnervated *and* devascularized nature upon creation. Thus, based on the volume of evidence documenting mechanisms that promote both nerve and muscle regeneration, we investigate the use of chronic FES to promote regeneration, reinnervation, and revascularization of regenerative muscle grafts.

The efficacy and long term effects of FES are strongly influenced by the design and biocompatibility of the stimulating electrode. Designs are optimized to meet the desired contact surfaces, magnitude of signal, and implantation method (30). In general, the selectivity and specificity of stimulation rises in parallel with the invasiveness of implantation (30). By implanting closer to muscle and neural structures, a more direct connection can be made to individual fibers for stimulation/recording purposes at the expense of iatrogenic injury from surgical implantation. For their high selectivity and specificity, epimysial electrodes, which lie flat against the muscle surface, are commonly used for electrical stimulation in the context of therapy (31, 32), and recording for myoelectric prosthetic control (11, 12, 30, 33). Epimysial electrodes offer signal-to-noise ratios in the range of 4.5–6.5 for electromyographic recording (34–36). Epimysial electrodes cause less damage to the muscle fibers and exhibit robust durability because they are implanted directly onto the muscle in contrast to intramuscular electrodes which pierce the epimysium and rip through myocytes (30).

The biocompatibility of implanted electrodes poses a major concern (30). Implantation of the foreign body incites an immune response which often poses a challenge to the electrode's long term functionality and the tissues' health. Myocytes adjoining the surface of the electrode are chronically exposed to high currents and undergo detrimental morphologic changes (37). Moreover, the mechanical mismatch of electrode materials and soft tissue often causes aggregative inflammation at the implantation site which exacerbates the foreign body response (37). Over time, implanted devices may become isolated within a thick fibrotic capsule, drastically reducing the functional capabilities of electrical signal transfer for both stimulation and recording (38). For applications such as those in the field of neuroprostheses, inflammation caused by the foreign body response can preclude the required longevity (39).

Thus, with the considerations of the mechanical properties of muscle, biocompatibility, and tissue selectivity, we designed an epimysial electrode comprising of a polyimide film encasing hard, patterned, conductive, thin-film metals. Polyimide (PI) is a biocompatible alternative to silicone (40, 41), commonly used in biosensors and neural arrays (37). Its biocompatibility has been tested using level I and level II assays defined by the National Institutes of Health's *Guidelines for Blood-Material Interactions*. It has demonstrated high viability as an implanted material based on insignificant levels of cytotoxicity and hemolysis. This polyimide film-based electrode platform has been successfully used in prior studies for stimulating neuromuscular tissues without any major biocompatibility or foreign body concerns (42).

**Figure 1:**
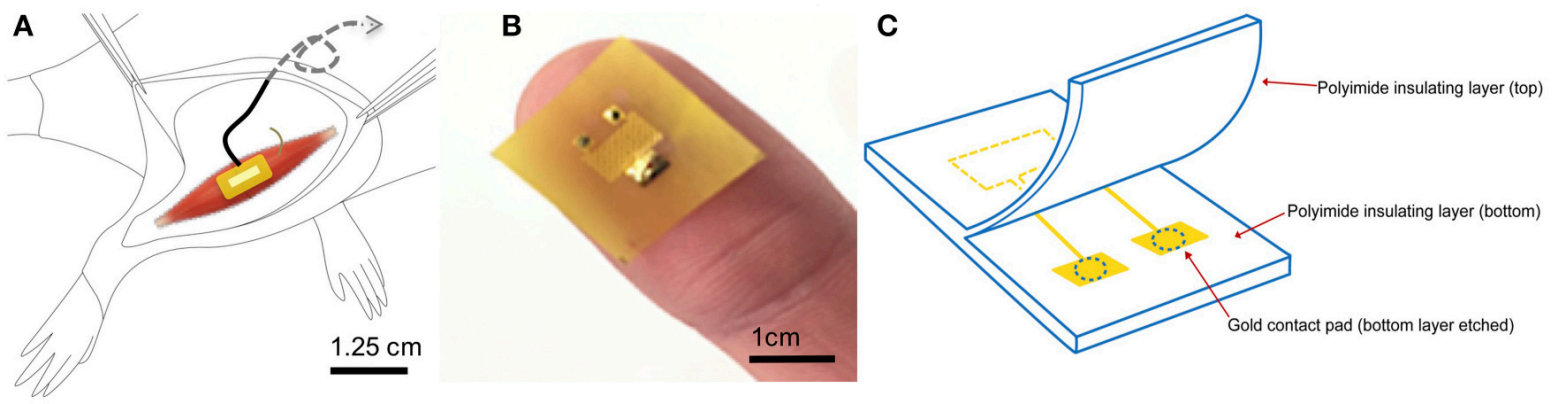
Electrode design and surgical implantation **(A)** Electrode is implanted epimysially on a free muscle graft created from the extensor digitorus longus on top of the biceps femoris. Wires are placed in a stress-release loop and tunneled subcutaneously up the back to the neck region. **(B)** Photograph shows the patterning and placement of contacts on electrode surface. **(C)** Schematic and components of the polyimide electrode. Muscle contacts the gold conducting surface through etched pads in the polyimide insulating layer of the electrode.

In this murine study, we explore the effect of chronic stimulation via a PI-based epimysial electrode on the regeneration, reinnervation, and revascularization of a regenerative free muscle graft (deinnervated and devascularized) that is neurotized during surgery to form a peripheral nerve interface (Figure 1A). We hypothesize that electrical stimulation will decrease the time course of reinnervation, improve revascularization, and result in muscle grafts capable of producing stronger EMG signals. Following surgical creation of a neurotized free muscle graft we implant the epimysial electrode. We study the effort of stimulated muscles (*n* = 6) against controls in which an electrode was implanted, but no stimulation is applied (*n* = 6) using electrophysiological and histological tests over the course of 40 days. We compared these to established cases in which no electrode was implanted.

## Methods

### Fabrication and Characterization of Electrode

The fabrication of the electrode has been described in detail in McAvoy et al. (42). Briefly, titanium and gold were deposited onto polyimide films (10 **μ**m diameter, PI 2574 from HD Microsystems) in a serpentine pattern to create a large area of conductivity (Figure 1B). The thickness of the gold and titanium layers were 500 and 10 nm, respectively (Figure 1C). The electrode advantageously offers low electrode impedance and high charge injection capacity due to a large electroactive contact area of 1.25 mm. The size of the electrode was designed to be 1 cm^2^, accommodating the full width of the muscle. Flexible polyimide was chosen for its compliant nature and ability to match the mechanical properties, as well as wrap snugly around the curvature of the muscle, which is important for biomaterial performance (43). Electrode integrity in biological milieu was previously tested and shown to be stable to large impedance changes and delamination (43). The electrode was then attached to the lead wires using solder or PI tape.

We characterized the mechanical properties of the electrode using an Instron machine (Instron 5943 single column tensile tester). Specifically, under a tension test, with the grips pulling at a speed of 0.02 mm/s, dry and wet PI electrodes were elongated to fracture. The Young's modulus was calculated using the resulting values from five independent trials.

### Surgical Implantation

All animal experiments were conducted under the supervision and approval of the Committee on Animal Care at the Massachusetts Institute of Technology (MIT) on Lewis rats (*n* = 12, weight range: 160–188 g) under 1–2.5% isoflurane anesthesia. Animals were randomly assigned to a control (*n* = 6, no stimulation) or experimental group (*n* = 6, stimulation). In all animals, an incision was made following the line of the tibia on the right distal hind limb. A free muscle graft was created by harvesting, deinnervating, and devascularizing the extensor digitorus longus (EDL). The common peroneal (CP) nerve was then distally transected and brought up to the superficial fascia of the biceps femoris (BF) through a slit in the BF. The EDL was secured to the fascial layer of the biceps femoris and neurotized using the CP. The PI electrode was sutured over the EDL muscle graft. Wires were placed in a stress-release loop to allow for movement without tugging on the implant and tunneled to a head cap. Skin was then closed with 4–0 Nylon suture, wound glue and wound clips. Wound clips were removed at post-operative day 10. Weekly weight measurement and physical evaluation were performed to monitor for infection and other negative sequelae resulting from surgery.

### Stimulation Parameters

Stimulation experiments were performed starting one week after the initial surgery to allow for inflammation to subside. Animals implanted with an electrode were stimulated thrice weekly with 40 Hz biphasic square wave pulses for 45 min. Between 2 and 5 mA of current was delivered depending upon the threshold required for strong muscle activation, as inspected through palpation. This stimulation range has been found to enhance functional recovery following nerve transection and/or denervation of the hind limb muscles in murine models (20, 44, 45). In our pilot experiments, these stimulation parameters consistently activated muscles while minimizing harmful effects.

### Insertional EMG Recording

Starting 10 days after surgery, insertional needle electromyography (EMG) measurements were obtained using a 30G monopolar needle electrode placed in the muscle graft every 10 days. A ground electrode was inserted into the subcutaneous space on the back. Electrodes were adjusted to eliminate noise or moving baselines before recording. EMG signal was recorded for three separate 1-min segments taken from three distinct areas in the graft. Care was taken to avoid the regions near the electrode.

### Electrophysiological Testing

To perform electrophysiology, an incision was performed to isolate the CP and muscle graft. A pair of 30G bipolar electrodes were placed in the muscle graft and attached to a stabilizer to prevent motion. A hook electrode was fabricated by stripping 0.5 cm of insulation from a wire structured in a hook shape. Drops of mineral oil were placed on the cradle holding the nerve in the hook electrode to insulate the connection. Stimulation of varying amplitudes and frequencies were delivered and resulting potentials were recorded from multiple (3+) locations within each muscle. Quantitative parameters (duration, amplitude, area, area/amplitude, size index, phases, and turns) were determined using a semi-automated custom MATLAB program. This testing was performed during a terminal surgery at 4.5 weeks after the initial implant of the electrode.

### Histology

During a terminal procedure, the entire region consisting of the implanted electrode and regenerative muscle graft, including the innervating nerve, was collected and fixed in 4% paraformaldehyde. After 24 h, it was paraffin processed, embedded, and sectioned at 5 um. Four sections from each animal were stained with hematoxylin and eosin (H&E) to investigate the morphology of tissues. Revascularization was primarily assessed through the presence of neovasculature and angioblasts. Mason's trichrome and toluidine blue stains were used to assess collagen and connective tissue deposition, integration of electrode material, foreign body reactions, and the presence of mast cells.

### Statistical Analysis

Results between the control and experimental groups were compared using a student's unpaired *t*-tests.

## Results

**Figure 2:**
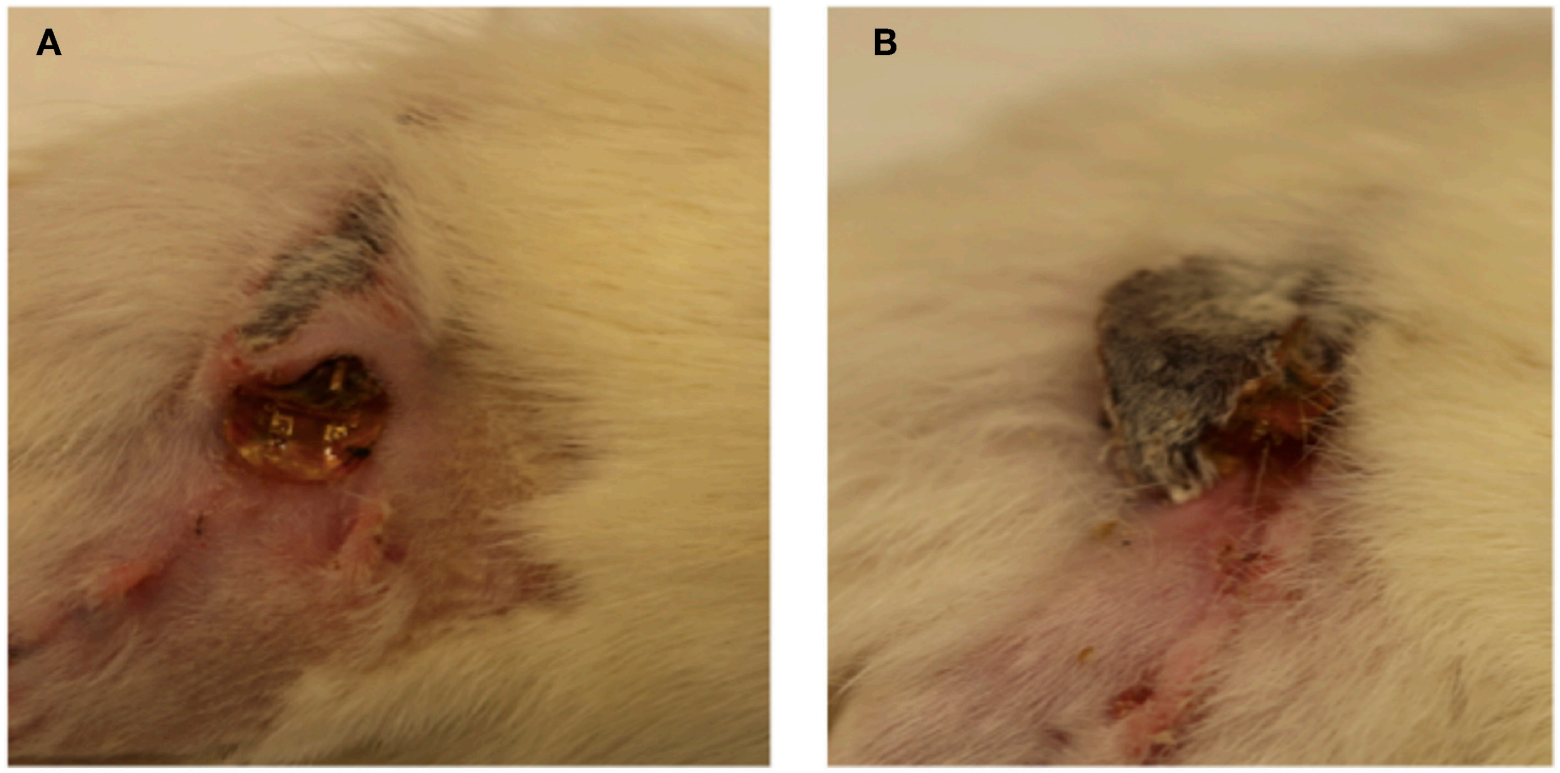
**(A,B)** Implant extrusion: 2 weeks after surgery, electrodes had extruded from the site of implantation in two animals. While the skin had healed fully around the electrode in both cases, infection was present in one case **(A)**.

After surgery, 10 out of the 12 animals recovered without incident. Two animals in the stimulated group demonstrated rejection of the subcutaneous implant via large exposure and extrusion of implant through the skin (Figure 2). This may have been due to the behavior of the rat as a result of irritation, which enabled the migration of the electrode through subcutaneous fascia and through the skin. Full healing of the skin occurred around the extruded portion in both cases (Figure 2), however an active infection was present in one animal (Figure 2A). Revision surgery was performed to address wound dehiscence caused by the extrusion of the implant. Any sharp edges were trimmed and made smooth before reimplantation. Close monitoring demonstrated integration and no further extrusion. Thus, these two animals were included in the experimental cohort for further testing.

A layer of facial and scar tissue encapsulated the electrode's wire as it tracked up the back of the animal in the subcutaneous space, providing a lubricious tract for sliding of the wire during locomotion. Grafts were harvested between 40 and 50 days post-operation and demonstrated 50–70% atrophy in both groups. In the experimental and control groups, grafts were on average 53 ± 8% and 62 ± 4% of the original, respectively. This was not a statistically significant difference (*p* = 0.42, unpaired *t*-test,). This rate of atrophy in the grafts is consistent with prior studies in which no electrode was implanted onto the grafts (9).

### Time Course of Reinnervation Is Delayed

**Figure 3:**
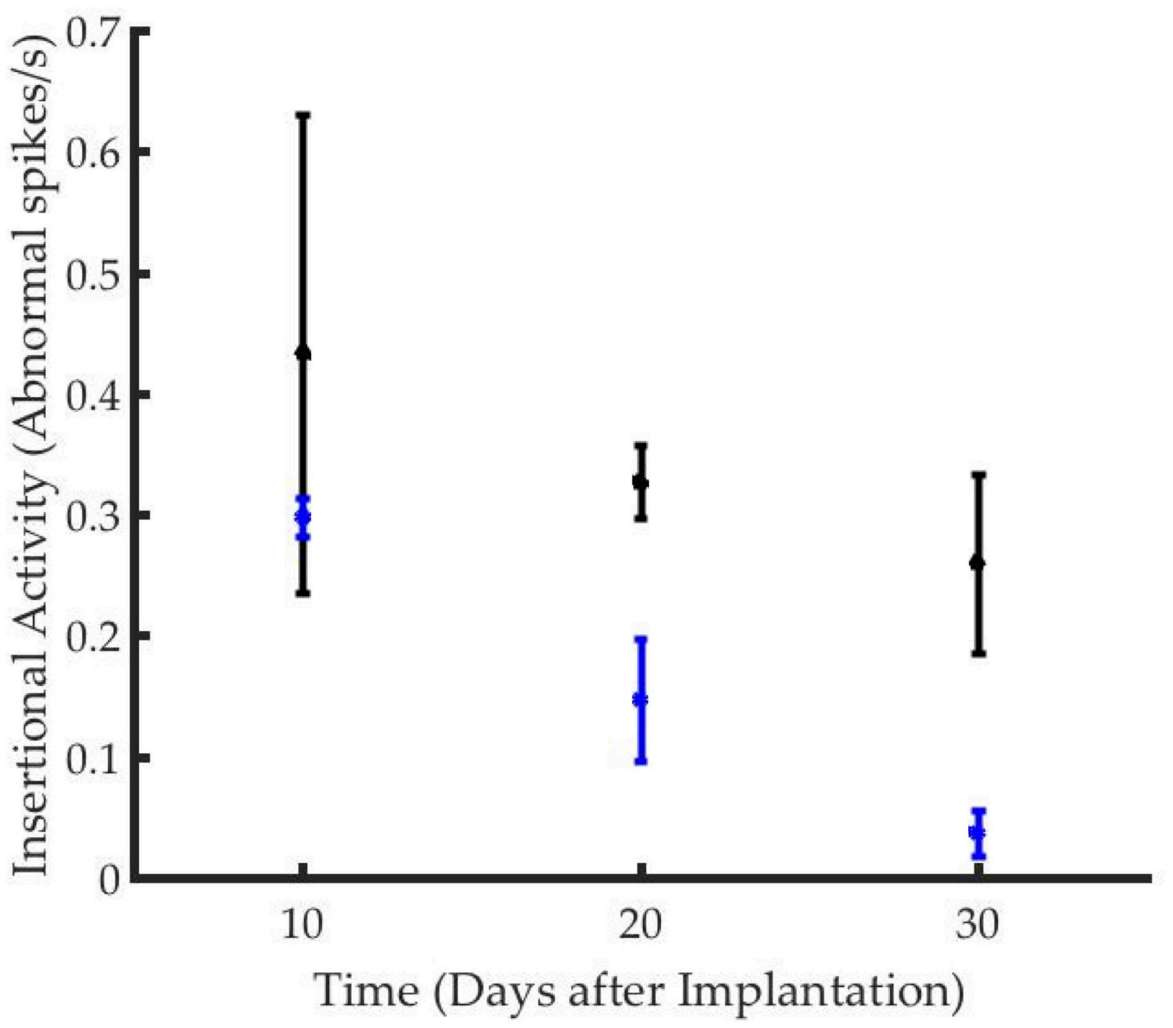
Graft reinnervation in the stimulated group (black) was delayed as compared to unstimulated controls (blue).

The time course of reinnervation of regenerative muscle grafts has been previously studied using insertional needle EMG (9, 11). While healthy innervated muscles are generally “electrically silent,” deinnervated muscles demonstrate frequent, spontaneous depolarization (9, 46). The frequency of waves types including fasciculation potentials, myokymic, or neuromyotonic discharges, and end plate spikes, are used to quantify the rate of reinnervation (46). While the frequency of abnormal activity declined in both groups over time, the time course of reinnervation for animals in the stimulated group significantly exceeded that of the control group (Figure 3). The frequency of insertional activity at 20 and 30 days was significantly different from that of the control group (*p* = 0.01, student's unpaired *t*-test). Unstimulated controls demonstrated a reinnervation time course that was consistent with previously reported timelines for devascularized and deinnervated free muscle flaps (14, 47). Notably, numerous positive sharp waves and fibrillation potentials were observed in the recordings from stimulated animals, which were indicative of incomplete reinnervation.

### Histological Results Demonstrate Inflammatory State

**Figure 4:**
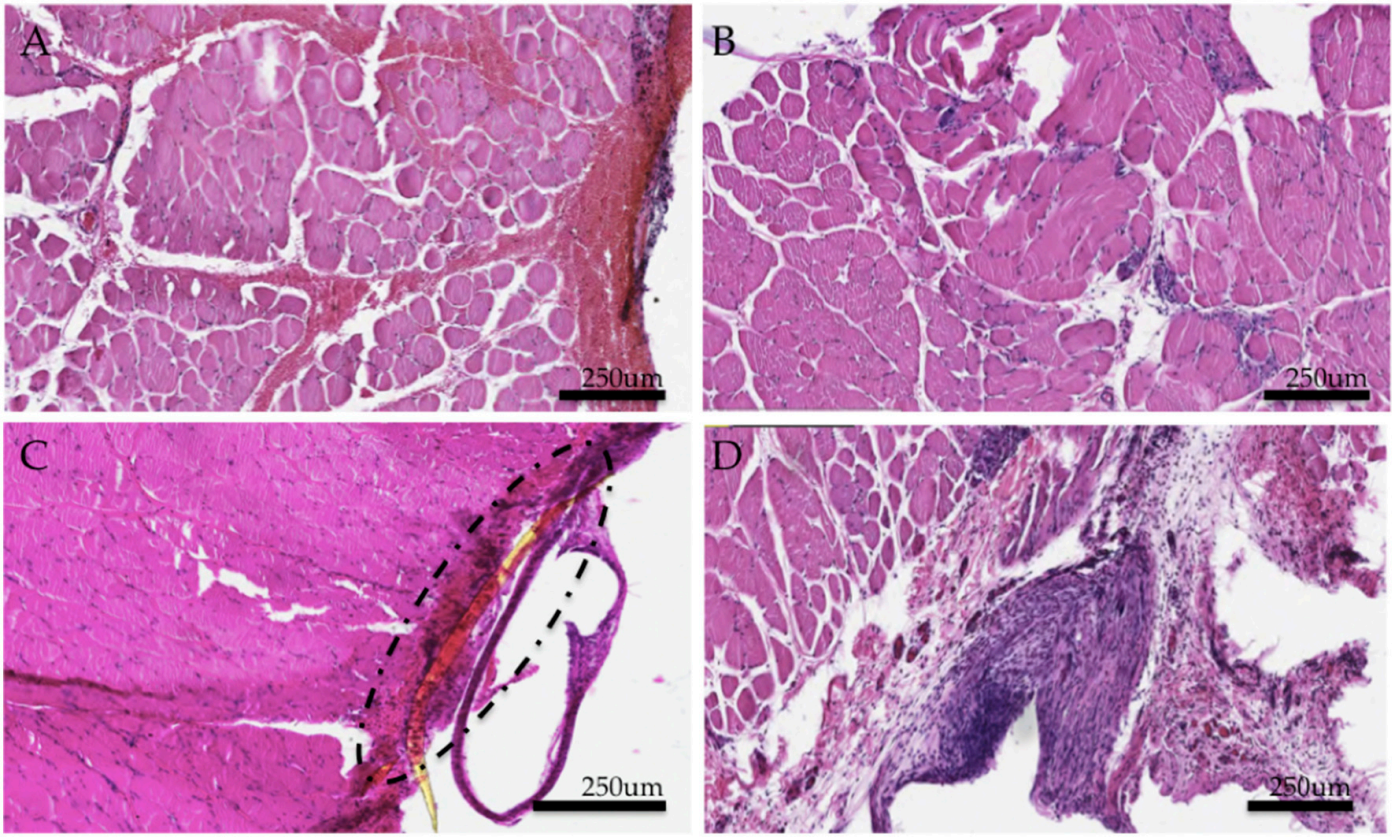
Histological study of experimental muscle grafts. **(A)** Perimysial inflammation found in cross sections **(B)** Neutrophilic infiltrate and early necrotic fibers **(C)** Area of implant and surrounding fibrotic capsule around a fragment of the PI electrode **(D)** Region encapsulating the implant (removed here) demonstrates an active inflammatory response and a thick fibrotic capsule. Numerous muscle fibers demonstrate necrosis. All tissues were representative samples from the experimental group stained with hematoxylin and eosin.

Hemotoxylin and eosin (H&E) staining of the tissues demonstrated a thick capsule forming around the implanted PI material (Figure 4C), consistent with a standard scar tissue formation process and biocompatibility response to a foreign material. Chronic perimysial inflammation was visualized around groups of fibers as seen in Figure 4A. Further, neutrophilic infiltrate and early necrosis via macrophage-mediated tissue necrosis were seen between muscle fibers at the 6 week time point (Figures 4B,D). When compared to controls, staining of experimental grafts showed a degeneration of muscle tissue and fewer regenerating myocytes, consistent with a chronic inflammatory state. Muscle sections appeared partially revascularized through several small-diameter vessels and angioblasts found at the periphery.

### Electrophysiology Suggests Myopathy

Our electrophysiological investigation comprised of a comparative analysis of the morphology (polyphasia, duration, and amplitude), stability and firing characteristics of over 300 motor unit action potentials (MUAPs) collected under a variety of stimulation parameters at 4.5 weeks after implantation. These action potentials represent the extracellular compound potentials of the muscle fibers of a motor unit.

**Figure 5:**
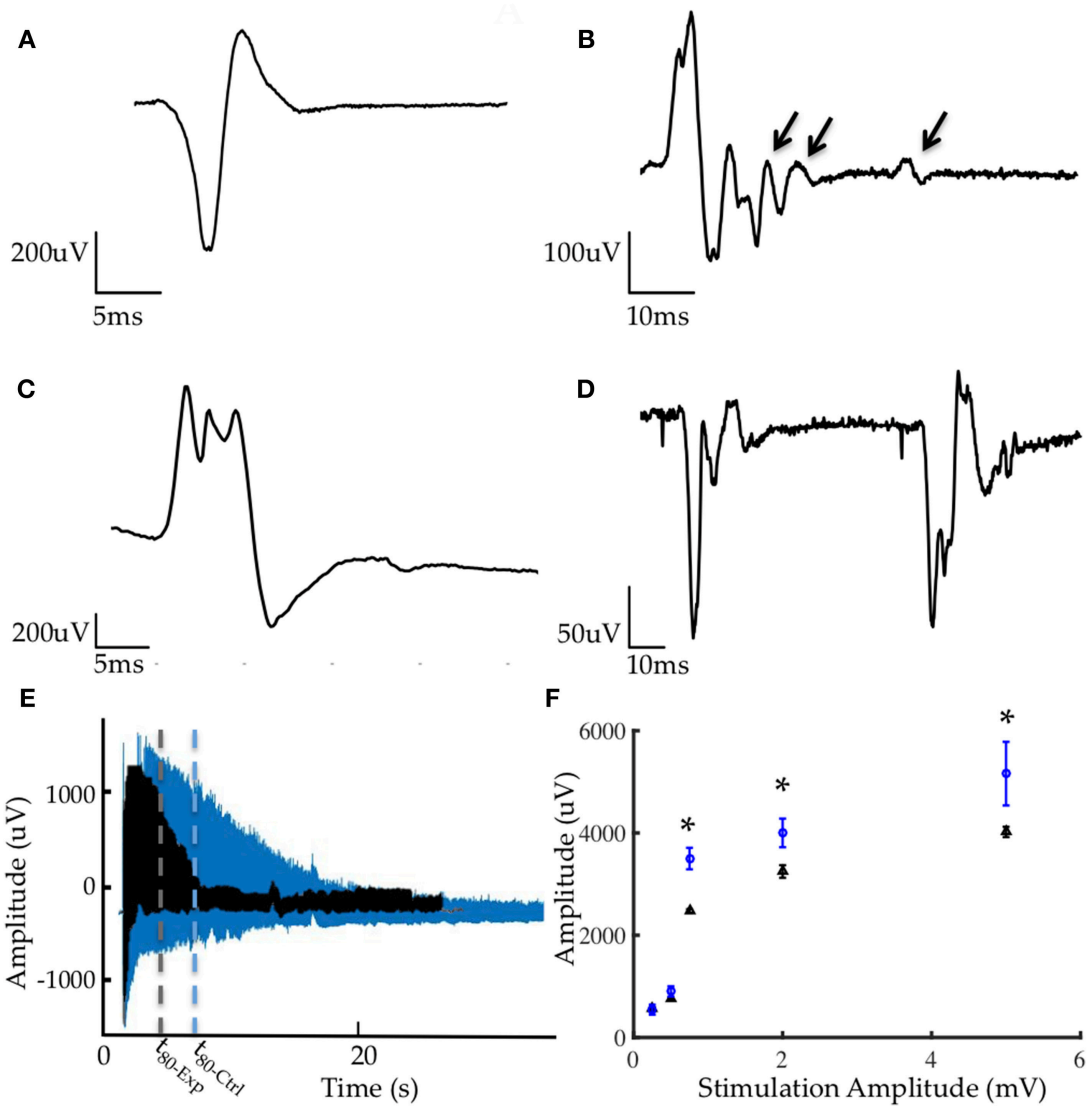
Representative electrophysiology **(A)** MUAP from control graft **(B)** MUAP from experimental graft demonstrating polyphasia from asynchronous firing and satellite potentials (denoted by arrows) generated from motor units early in reinnervation **(C)** MUAP from experimental group with serrations and elongated pulse width **(D)** Lack of stability in EMG morphology resulting from myopathic fibers in the experimental group. **(E)** Comparison of fatigue under tetanic contraction (40 Hz stimulation) between control (blue) and experimental (black) groups demonstrating greater fatigability in the experimental group. t_80_ denoted by dotted lines represents the time required for the muscle to reach 80% of its initial EMG amplitude. **(F)** Average CMAP amplitudes from all animals in the experimental (blue) and control (black) groups, with results at 1, 2, and 5 mA being statistically significant (^*^p < 0.001).

MUAPs from the control group presented with normal morphologies, pulse widths, and amplitudes comparable to prior studies (Figure 5A) (9, 11). MUAPs in the stimulated group were significantly more polyphasic (*p* < 0.0001, student's unpaired *t*-test) and demonstrated frequent satellite potentials (Figure 5B). Polyphasia is often a result of asynchronous firing and satellite potentials are generated in motor units early in the reinnervation or necrotic processes. Additionally, over 80% of potentials generated at low stimulation amplitudes yielded pulse durations between 8 and 18 ms, which is uncharacteristic for healthy myocytes (Figure 5C). Finally, MUAPs from the experimental group were unstable and presented variation in morphology from potential to potential (Figure 5D).

Fatigability was quantified through the fatigue time constant, t_80_, representing the length of time required for the muscle to reach 80% of its initial EMG amplitude under a tetanized contraction (40 Hz stimulation). In this study, grafts in the stimulated group fatigued significantly sooner than those in the non-stimulated group (Figure 5E). The average t_80_ for the experimental group was 10.07 ± 4.07 s and 37.09 ± 7.29 s for the control group.

The compound muscle action potential (CMAP) was measured in response to 0.25, 0.5, 1, 2, and 5 mA stimulation via a hook electrode on the common peroneal nerve. Peak-to-peak amplitudes for the non-stimulated group were significantly higher than those measured in the stimulated group at 1, 2, and 5 mA (Figure 5F) suggesting better reinnervation and healthier myocytes.

### Characterization of Mechanical Properties

We performed a comparison of wet and dry PI to identify changes in the compliance due to its environment. The material properties of the PI electrode and muscle tissue were measured using an Instron machine. The Young's modulus of the PI material when dry was measured to be 3.74 ± 0.30 GPa. After soaking for 24 h in PBS, PI's modulus measured 3.72 ± 0.80 GPa, which was not significantly different from its dry nature. These values fall within the general range reported in literature (10, 13). The modulus of the extensor digitorus muscle harvested from animals yielded a young's modulus of 44.3 ± 20.5 MPa. While the PI material had a significantly higher young's modulus, its thin and flexible nature allowed it to curve with the muscle and make strong contact.

## Discussion

Given the recent advancements in microsurgical technique catalyzed by technological developments in surgical instrumentation, there is increased use and interest in regenerative free muscle grafts. These grafts can be used in reconstructive procedures to provide soft tissue coverage and serve as a source of electromyographic signals for neuroprosthetic control. However, the ultimate functionality of regenerative free muscle grafts is dependent on the extent and time course of reinnervation, preservation of muscle mass, and vascularization. Optimization of regeneration is critical, especially in the case of peripheral nerve interfaces and reconstructive surgical procedures where the signal quality and biomechanical functionality are the primary goals (9, 48).

Electrical stimulation has been demonstrated to promote the regenerative processes of devascularized *or* deinnervated muscle (20, 23, 45). In this study, we investigated the effect of chronic stimulation, using a polyimide electrode, on the regeneration of a free muscle graft, which was *both* devascularized and deinnervated. For the methodologies used herein, our results do not support our hypothesis that electrical stimulation delivered through an implanted epimysial electrode improves the regeneration of the muscle grafts.

### Implant Rejection by Host

Despite being comprised of biocompatible materials, the implanted electrode was extruded and caused wound dehiscence in two cases in the stimulated group. This PI electrode has been used in more than 20 animals for a variety of studies and its higher rejection rate in stimulated animals suggests that electrical stimulation may 1) lead to chronic irritation that prevents integration with the myoneural construct and 2) provide an impetus to explant the foreign body by the animal.

### Chronic Inflammatory State

In stimulated animals, histological evidence indicated an active, chronic, inflammatory response, which resulted in neutrophilic infiltrate, necrotic fibers, limited axonal sprouting, and perimysial inflammation. Normally, necrotic fibers in regenerating muscles are replaced by myotubes during the second week of regeneration (6). However, in this study, anisotropic myocytes and necrotic fibers were present even at the 6-week time point. These structural abnormalities likely contributed to the poor electrophysiological function observed.

### Delayed Reinnervation and Partial Myopathy

The electrophysiological data were suggestive of incomplete and delayed reinnervation and partial myopathy. Needle electromyography demonstrated a longer time course for reinnervation of stimulated grafts when compared to unstimulated grafts or those on which no electrode was implanted (9, 11). Nascent motor unit potentials of shorter duration and smaller amplitude were recorded in both groups (Figure 5), which is characteristic of reinnervating motor units. In grafts stimulated by the implanted electrode, abnormal and unstable MUAP morphologies, decreased CMAP amplitudes and increased fatigability were observed. These significant abnormalities in EMG and recruitment of fibers evidenced myopathic motor units in the stimulated experimental grafts. Corroborating with other data, incomplete healing and regeneration of the grafts, combined with the observed inflammation suggested a chronic myopathic state (46, 49).

Contrary to the accelerated regeneration expected as a result of electrical stimulation (through the upregulation of angiogenic and neurogenic growth factors), the PI-based electrical stimulation was detrimental to graft reinnervation and overall health. Regeneration of free muscle grafts demands an active revascularization and reinnervation process to supply essential nutrients, prevent disuse atrophy, and repopulate the necrotic core with myogenic precursor cells. Electrical stimulation may have interfered with the following key mechanisms that dictate the early stages of graft regeneration.

(I) Vascular beds are disrupted during surgery and a direct blood supply is not immediately available to the grafts. Hence, the revascularization of regenerative grafts occurs through four phases: (1) plasmatic imbibition, (2) inosculation, (3) capillary ingrowth, and (4) formation of larger vessels. During the first two phases, nutrient exchange depends primarily on diffusion. By monopolizing nearly half the superficial surface area of the muscle graft, the electrode may have impeded capillary ingrowth preventing rapid revascularization of the grafts, a critically important process (2) for regeneration. Stimulation likely exacerbated this process by causing unnatural micromotions of settling angiogenic cells during each contraction, impeding capillary ingrowth, and the formation of larger vessels.

(II) The stimulation parameters used in this study were intended to elicit muscle contractions (23, 44, 45). Given their deinnervated state, these muscles required a greater magnitude of stimulation for activation (50), stemming from the lack of motor neuron fibers distributing and amplifying the potential at neuromuscular junctions. This stimulation amplitude was likely too strong for regenerating nerve fibers, and may have contributed to the delayed and incomplete reinnervation (50) that was observed.

(III) The stiffness of the PI electrode exceeded that of the muscle tissue by an order of magnitude. Despite this mismatch in compliance, the electrode has functioned effectively in the past and with minimal negative consequences in this study. Nevertheless, under electrical stimulation, in the absence of full adherence and movement with the underlying tissue, contraction of the muscle would have resulted in sliding between the epimysium and the electrode. Due to friction at this interface, the electrode may have irritated the graft and provoked active foreign body and fibrotic response (51).

Given the results and considerations presented in this study, it is important to balance the known regenerative benefits of electrical stimulation through implanted electrodes with the demonstrated disadvantages caused in the acute inflammatory and regeneration phases. For devascularized free muscle grafts, it may be beneficial to implant devices 1–2 weeks after surgery to allow capillary ingrowth before covering the surface area through which it occurs. Alternatively, intramuscular electrodes, which are fabricated from fine wires and do not cover the surface of the muscle, can be utilized for this application. Importantly, stimulation should only commence once basic vasculature has formed to prevent disruption of integral angiogenic processes. Future work should optimize stimulation parameters to accommodate the specific needs of devascularized and deinnervated free muscle grafts. Overall, this study provides insight on electrical stimulation and the biocompatibility of polyimide epimysial electrodes for free muscle grafts in the early regenerative phase. In the development and implantation of electrode hardware that interfaces to free muscle grafts, we feel the electrode design and timing of electrode implantation are considerations of paramount importance.

## Data Availability

The data and code from this study can be obtained from the researcher upon reasonable request.

## Author Contributions

SS performed surgical implantation, data collection, analysis, and preparation of the manuscript. KV performed implant fabrication and data collection. MM assisted with the experiment conceptualization and preparation of the manuscript. PC helped with surgical implantation and data collection. OK assisted with fabrication of the implant. RL, DA, and HH assisted in manuscript preparation.

### Conflict of Interest Statement

The authors declare that the research was conducted in the absence of any commercial or financial relationships that could be construed as a potential conflict of interest.
